# Coronavirus (SARS-CoV-2) Pandemic: Future Challenges for Dental Practitioners

**DOI:** 10.3390/microorganisms8111704

**Published:** 2020-10-31

**Authors:** Ioana Roxana Bordea, Edit Xhajanka, Sebastian Candrea, Simion Bran, Florin Onișor, Alessio Danilo Inchingolo, Giuseppina Malcangi, Van H Pham, Angelo Michele Inchingolo, Antonio Scarano, Felice Lorusso, Ciro Gargiulo Isacco, Sergey K Aityan, Andrea Ballini, Gianna Dipalma, Francesco Inchingolo

**Affiliations:** 1 Department of Oral Rehabilitation, Faculty of Dentistry, Iuliu Hațieganu University of Medicine and Pharmacy, 400012 Cluj-Napoca, Romania; 2 President of Dental School, Medical University of Tirana, Rruga e Dibrës, 1001 Tirana, Albania; editxhajanka@yahoo.com; 3 Department of Pedodontics, County Hospital Cluj-Napoca, 400000 Cluj-Napoca, Romania; 4 Department of Maxilofacial Surgery and Implantology, Faculty of Dentistry, Iuliu Hațieganu University of Medicine and Pharmacy, 400012 Cluj-Napoca, Romania; dr_brans@yahoo.com (S.B.); florin.onisor@gmail.com (F.O.); 5 Department of Interdisciplinary Medicine (D.I.M.), University of Medicine Aldo Moro, 70121 Bari, Italy; ad.inchingolo@libero.it (A.D.I.); giuseppinamalcangi@libero.it (G.M.); angeloinchingolo@gmail.com (A.M.I.); giannadipalma@tiscali.it (G.D.); francesco.inchingolo@uniba.it (F.I.); 6 Nam Khoa Laboratories and Pham Chau Trinh University of Medicine, Hoi An 70000, Vietnam; phhvan.nkbiotek@gmail.com; 7 Department of Medical, Oral and Biotechnological Sciences, University of Chieti-Pescara, 66100 Chieti, Italy; antonio.scarano@unich.it (A.S.); drfelicelorusso@gmail.com (F.L.); 8 Director of Research at Human Stem Cells Research Center HSC, Ho Chi Minh 70000, Vietnam; drciroisacco@gmail.com; 9 Associate Professor of Embryology and Regenerative Medicine and Immunology at Pham Chau Trinh University of Medicine, Hoi An 70000, Vietnam; 10 Visiting Professor of Regenerative Medicine and Metabolic Disorders at Department of Interdisciplinary Medicine (D.I.M.), University of Medicine Aldo Moro, 70121 Bari, Italy; 11 Director of Multidisciplinary Research Center, Lincoln University, Oakland, CA 94102, USA; aityan@lincolnuca.edu; 12 Department of Biosciences, Biotechnologies and Biopharmaceutics, Campus Universitario “Ernesto Quagliariello” University of Bari “Aldo Moro”, 70125 Bari, Italy; andrea.ballini@uniba.it; 13 Department of Precision Medicine, University of Campania“Luigi Vanvitelli”, 80138 Naples, Italy

**Keywords:** SARS-CoV-2, COVID-19 pandemic in dental practice, COVID-19 risk assessment in dentistry, coronavirus disease, ACE2 receptor, Flügge’s droplets, MAS superior standard hepa filter

## Abstract

In the context of the SARS-CoV-2 (Severe acute respiratory syndrome coronavirus 2) pandemic, the medical system has been subjected to many changes. Face-to-face treatments have been suspended for a period of time. After the lockdown, dentists have to be aware of the modalities to protect themselves and their patients in order not to get infected. Dental practitioners are potentially exposed to a high degree of contamination with SARS-CoV-2 while performing dental procedures that produce aerosols. It should also be noted that the airways, namely the oral cavity and nostrils, are the access pathways for SARS-CoV-2. In order to protect themselves and their patients, they have to use full personal protective equipment. Relevant data regarding this pandemic are under evaluation and are still under test. In this article, we made a synthesis about the way in which SARS-CoV-2 spreads, how to diagnose a novel corona virus infection, what the possible treatments are, and which protective personal equipment we can use to stop its spreading.

## 1. Introduction

In December 2019, an outbreak of pneumonia appeared in Wuhan City. Wuhan is an important international trading centre in central China. This pathology was concluded to be generated by a novel Coronavirus (nCoV-2019). Since then, the virus infection has spread throughout the world, it has been declared a pandemic by WHO on 12 March 2020 [1,2,3]. It seems that the first COVID-19 (coronavirus disease 2019) cases were connected to a large fish and living animal market in this large metropolis. It was thought that the path of direct transmission came from a food market. Since then, person-to-person transmission has been found be one of the main spreading mechanisms of COVID-19 [1,2,3].

After the identification of the initial cases, the pandemic hit almost all the nations in the world. Now, there are more than 1,113,307 deaths worldwide due to the coronavirus pandemic. The updated data of Johns Hopkins University identified 1,113,307 deaths. On the other hand, 39,964,414 contagions are global. COVID-19 has spread to 189 countries and territories and there are approximately 39,964,414 confirmed cases (as of 19 October 2020) [4].

**Figure 1:**
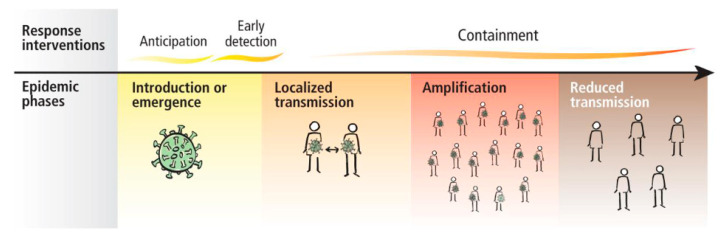
Operational considerations for case management of COVID-19 in health facility and community-Interim guidance 19 March 2020 [3].

The WHO (World Health Organization) presented the guidance for case management of COVID-19 in health facility and community Interim on 19 March 2020 [3]. The response interventions proposed by the WHO are presented in Figure 1.

Because this pandemic emerged in our lives and has produced a lot of changes, dental professionals have to introduce new strategies to perform dental treatments in order to reduce the risk of cross infection. A study performed by a team of Jordanian dentists showed that dental practitioners have very little information regarding the measures they have to take in order to protect themselves and their patients [5]. In his study, Ing showed that 4% of deaths were dentists because of the lack of protection equipment [6].

In this article, we made a synthesis about the way in which SARS-CoV-2 spreads, how to diagnose a novel corona virus infection, what the possible treatments are, and which protective personal equipment we can use to stop its spreading.

### 1.1. Epidemiology

**Figure 2:**
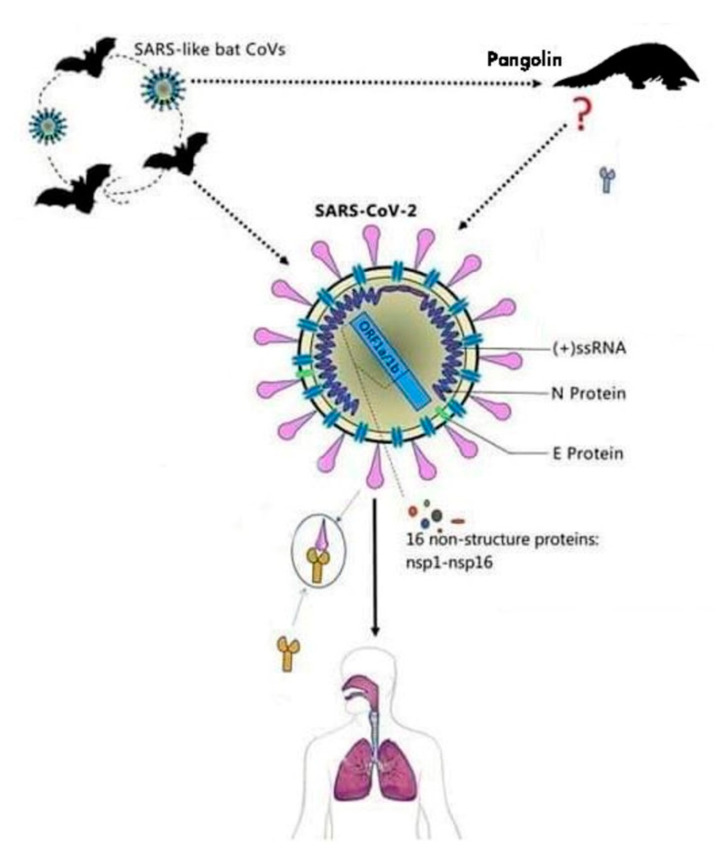
Conformation of SARS-CoV-2 (figure drawn by Giovanna Dipalma).

The first name given to this virus was 2019-nCoV, after a short period of time the name of the virus was changed due to the similarity with the SARS virus into SARS-CoV-2 [7]. The virus comes from the family of Coronaviridae and is made of single stranded RNA viruses [7]. This virus can be secluded from animal species and can determine cross infection, passing the barriers of certain species and infecting animals and humans. The virus has a cover that is composed of glycoproteins that look similar to a solar crown, as shown in Figure 2 [7].

In the literature, there are four genera of Coronaviruses. Two of the genera, γ-CoV and δ-CoV, determine changes in birds, while the other two genera, α-CoV and β-CoV, contaminate mostly mammals and also humans, by determining changes in different systems of the organism like the respiratory, gastrointestinal, and central nervous systems [7,8,9,10,11]. The new virus that determined infections in Wuhan belongs to the β-CoV family of viruses that includes the SARS-CoV (Severe Acute respiratory syndrome coronavirus) and MERS-CoV (Middle East respiratory syndrome), two viruses that are known for the infections they caused several years ago [8,9,10,11,12,13,14]. 

The nucleotide sequence similarity between SARS-CoV-2 and SARS-CoV is of about 80% and approximately 50% between SARS-CoV-2 and MERS-CoV. This could explain the reason why this novel virus is less deadly than the other two. Hence, its routes of transmission can spread the SARS-CoV-2 faster than the other two viruses [15,16,17,18,19,20]. It has been suggested that the natural host of SARS-CoV-2 may be the Rhinolophus affine bat, due to the similarities in the RNA (ribonucleic acid) sequence of the coronavirus found in the bat (96.2%) and SARS-CoV-2, and the intermediate host is the pangolin, with a genome sequence similarity of 99% between SARS-CoV-2 and the coronavirus found in these species [15,16,17,18,19,20].

As far as the intermediate guest is concerned, there are recent studies that contradict the hypothesis of appearance from the pangolin [21]. Some studies say that the first SARS-CoV-2 found in Wuhan’s HU-1 patient, in early December 2019, was perfectly adapted to humans, that is, despite being replicated several times in the following months, it did not undergo genomic transcription changes, remaining practically almost unchanged [17,18,19,20,21,22,23,24].

The bat is now considered to be the first source of the virus and the initial hypothesis of the infection from the pangolin that has been considered to be an intermediate host has been discarded [17]. Tang et al. suggested that the genomic sequence between the pangolin virus and the human one is not 99%, but lower (84%) than that the bat one (96%) [22].

Scientists are now taking into consideration the fact that the virus evolved and is infecting humans that were asymptomatic for three months, and in the same manner it increases the infectious capacity and reduces lethality [21].

This type of virus has the same structure as the common coronavirus, possessing the “spike protein” in the exterior structure of the envelope of the virion, and besides this, in its structure we can find proteins like nucleo, poly, and membrane proteins that include RNA polymerase, papain-like protease, helicase, 3-chymotrypsin-like protease, accessory proteins, and glycoprotein. The S protein from coronavirus can bind with the receptors of the host to encourage viral entry into target cells. Although there are four amino acid variations of S protein between SARS-CoV-2 and SARSCoV, the first can also bind with the human angiotensin converting enzyme 2 (ACE2), the same host receptor for the SARSCoV. SARS-CoV-2 cannot bind with cells without the presence of ACE2. The recombinant ACE2-Ig antibody, SARSCoV-specific human monoclonal antibody, and the serum from a convalescent SARS-CoV-infected patient can neutralize SARS-CoV-2, and thus confirms ACE2 as the host receptor for SARS-CoV-2. Due to the high affinity between ACE2 and SARS-CoV-2 S protein, it has been suggested that the population with a higher expression of ACE2 might be more susceptible to Coronavirus Disease 2019 (COVID-19) [17,18,25,26,27].

There is a great number of articles stating the fact that SARS-CoV-2 uses the S protein complex and the ACE2 receptor to entry the host cell. This information is used in different ways and domains, and above all in therapeutic treatment modality, diagnostic purposes, and infection transmission and prevention [28,29,30,31,32,33,34,35,36,37,38,39,40,41,42,43,44,45]. 

Zhou et al. indicated that the angiotensin-converting enzyme II (ACE2) is likely the cell receptor of SARS-CoV-2, which is also the receptor for SARS-CoV. Moreover, it has been proven that SARS-CoV-2 does not use other coronavirus receptors such as aminopeptidase N and dipeptidyl peptidase 4 [44]. Xu et al. showed that the S-protein of the SARS-CoV-2 supports a strong interaction with human ACE2. Those findings suggest that the ACE2 plays an important role in cellular entry of the SARS-CoV-2, thus, ACE2-expressing cells may act as target cells for susceptible to SARS-CoV-2 infection. Moreover, this research has shown that the ACE2 could be expressed in the oral cavity, which was highly enriched in the oral epithelial cells, especially at a higher level in the tongue than in the buccal and gingival tissues. These findings indicate that the oral mucosa can express a potential high-risk route of SARS-CoV-2 infection transmission [45]. This fact underlines the importance of dentists and dental healthcare workers to wear all the protective measures that are indicated to prevent infection [18]. 

The connection between the host and the virus is encouraged by the S glycoprotein that integrates the receptors of the host cells to produce the viral infection of the cells. This glycoprotein is part of the class 1 viral fusion proteins and it has more than 1300 amino acids [46].

**Figure 3:**
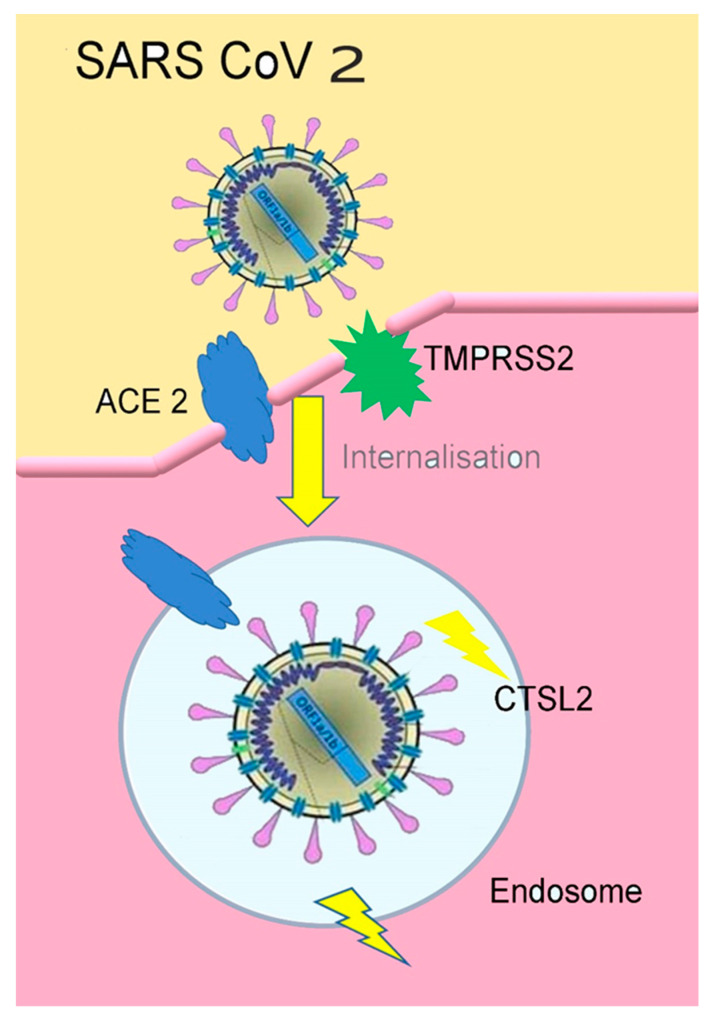
The connection of SARS-CoV2 to ACE2(figure inspired by https://www.the-scientist.com/news-opinion/receptors-for-sars-cov-2-present-in-wide-variety-of-human-cells-67496 [48], and drawn by Giovanna Dipalma).

The SARS CoV 2 can also bind a specific enzyme in the human body that is represented by the angiotensin-converting enzyme 2 (ACE 2), in order to bind the virus to the cells which need this enzyme [47]. The way the entire process takes place is presented in Figure 3. 

TMPRSS2(transmembrane protease serine 2) is an enzyme used by the SARS-CoV2 for S protein priming. CTSL2 (Cathepsin L2) is a gene, proteins encoded in this gene are members of the peptidase C1 family. For the SARS-CoV2 virus entering the human cells, Spike (S) protein needs to be cleaved by the cellular enzyme furin [49,50].

Furin is an enzyme, encoded by the FURIN gene, in the cells, belonging to hydrolases, splits proteins (inactive precursors) and transforms them into an active biological state (mature proteins) [44,45]. 

The S protein allows the virus to transfer the genome into the cell which leads to viral replication. In order to become active, the S protein must be cleaved by proteases. The S protein has two functional domains S1 and S2. S1 is implicated in the initial stage of viral entry, using its receptor binding domain to link to the receptors of the target cell and S2 acts in the second stage, fusing the cell and the viral membrane containing amino acid sequences necessary to continue the infiltration process [50,51,52,53,54].

In the corona virus family, the S protein has the largest variable amino acid sequence. In this virus the furin site is located between S1 and S2 subunits not unlike the pattern found in high-pathogenic influenza viruses, but not in other members of the Beta coronavirus genus [50,51,52].

Several patches of the RBD (receptor binding domain) are similar in SARS-CoV and SARS-CoV-2, an overall amino acid sequence identity of 76.47%. Because of the five important interface amino acid residues, four of these are different in SARS-CoV-2, and its S protein has a significantly higher binding affinity to human ACE2 than SARS-CoV S protein. Regardless of this, the two viruses shared an almost identical 3-D structure of the RBD which demonstrates similar Van der Waals electrostatic properties [50,51,52,53,54].

As treatment options, the effective antibodies against SARS-CoV’s spike S protein did not bind to the SARS-CoV-2 S protein. So, a vaccine sounds more tempting, but in order to create a live attenuated vaccine we must limit the replicative capabilities of the virus. In order to achieve this, we could remove the furin activation sequence which is essential for cell–virus fusion, thus allowing it to replicate. By doing so, the immune system can create antibodies in order to neutralise the virus and protect the body from further infections [50,51,52,53,54,55].

Another option would be to isolate antibodies from patients who have recovered from COVID-19 by using the novel S protein structure and mapping its structure in order to mass produce them [50].

In 2012 a new corona virus MERS-CoV (Middle East Respiratory Syndrome Coronavirus) was discovered resulting in a disease that manifests itself as severe respiratory disease with renal failure. The fatality rate was up to 38%. The place of emergencies was the Middle East, specifically countries where dromedary camels were identified as species harbouring the virus. Another outbreak was seen in 2015 in South Korea where the final tally was 36 people killed out of 186 confirmed cases. The SARS-CoV outbreak in southeast China had a worldwide tally of 774 killed from 8096 infected with a fatality rate of 9.6% [56].

### 1.2. Viral Load-Inflammation 

The world virus comes from Latin and means “poison”. Viruses are small microorganisms whose size varies from 0.2–0.3 μm to 1 μm. They need a host cell for living and reproducing (bacterial, vegetal, or animal). They have a very simple structure with an external cover of glycol proteins and lipids, called an envelope or pericapsid, in which there is a protective coat called capsid surrounding the virus genome. In the literature, there are DNA and RNA viruses, double stranded (DNA virus or dsRNA virus) or single (ssDNA virus or ssRNA) stranded. For the latter there is the “polarity” (consisting in process coding the virus) which can be positive or negative, namely ssDNA, ssRNA-, ssDNA+, or ssRNA+ (coronavirus) [57,58]. The cell replication (cytoplasmic or nuclear) is guided by the genome. The genome of the virus enters the host cell, and in few hours the formation of thousands of viral particles is performed, and they spread in the external environment. The replication of the RNA virus occurs easily with errors, as there is no RNA polymerase during the transcription. The high number of viruses as well as the error high frequency during the transcription are the main factors explaining the fast capacity to evolve proper of the SARS-CoV-2. Resistance to therapy is justified by the RNA mutation, even if it is very small, and allows the virus to avoid the attack of the immune system, continuing to change in terms of response in order to adapt to the constant changes of the genome [57,58].

Corona viruses are classified according to their own nature, structure, genome, and replication. The main feature of viruses is the infection of a special type of cell on which surface there are receptors which are similar to the binding. When binding with the host cell membrane is performed through those receptors, the virus penetrates the cell with its own genome, DNA or RNA. In this way. replication and multiplication of the virus starts. After the virus replication, the host cell usually dies. freeing new microorganisms in the surrounding environment where they can keep on infecting a new host cell having completed their lifecycle [59,60].

The Furin is considerably present in the lung tissue, in the intestine and the liver, this would make those organs as potential target of the 2019-nCoV infection. In dental field, Furin expression has been also revealed by the epithelium of the human tongue and in significant quantities in the squamous cell carcinoma. Researchers have shown a high availability of ACE2 receptors as well as the presence of Furin. Therefore, the tongue has a high risk of coronavirus infection and the SCC increases the risk in case of coronavirus exposure. The cleavage site on the spike similar to Furin plays an important role in spreading the 2019-nCoV virus [61,62,63,64,65,66,67].

At the moment there are some researches in which this site is eliminated, by observing effects or blocking the action of Furin, as issued on Nature [16]. This explains the strategic possibilities which we can sum up in this way:

**1.**  Molecules inhibiting Spike-ACE2.
**2.**  Anti-Serin protease molecules.
**3.**  Molecule inhibiting the HR1 domain (sub. S2).
**4.**  Inhibitors of viral enzymes, namely antivirals [16,68].

The activation of TMPRSS2 (Trans Membrane Protease, Serine 2) is fundamental as SARS-CoV-2 infects the lung cells, SARS-CoV-2 can use the TMPRSS2 to trigger the S protein. Some studies highlight that the TMPRSS2 is an important element of the host cell as it is essential for spreading a great number of viruses causing potentially significant infections, as the influenza A and coronavirus. Important data show that the TMPRSS2 is not necessary for the development and homeostasis and so it is potentially and sensible pharmacological target able to inactivate the infection. It is important to underline that the serine protease inhibitor, camostat mesylate, blocks the TMPRSS2 activity. This treatment, or something similar with likely increased antiviral activity (Yamamoto et al., 2016), could be used for treating patients with SARS-CoV-2 infection. Further studies suggest that the activation mediated by Furin on the S1 / S2 site within the infected cells could activate the subsequent access depending on the TMPRSS2 within the target cells [69,70,71,72,73].

An analysis on the real proteolytic elaboration of the protease on the S protein, and on its cleavage in S1 and S2 through detection with the antigenic system, underlined the existence of a band corresponding to the subunit S2 and protein S of the host cells infected by the virus of the vesicular stomatitis (VSV) containing SARS-2-S [61,62,63,64,65,66,67].

Knowing the action of the SARS-CoV-2 may allow to produce targeted drugs and vaccines against the COVID-19, a new treatment modality investigated is the one using PRP (platelet rich plasma), PRF (platelet rich fibrin), and CGF (concentrated growth factors) [74,75,76,77,78,79,80].

In 2011, a study on macaques infected by coronavirus with severe lung infections has been taken into consideration as the saliva droplets were source of infection [81].

It has been confirmed that the epithelial cells of the salivary glands covering the salivary ducts had high ACE2 expression (Angiotensin-Converting Enzyme 2), and therefore the first target cells have been revealed together with the first production source of virus [46,49,50,82,83,84,85].

The ACE2 expression in human organs has been analyzed by considering data collected by the portal Genotype-Tissue Expression. It is noted that the ACE2 expression in minor salivary glands was higher than the ones found in lungs. As a result, salivary glands are targets for SARS-CoV [82].

Another confirmation derives from the fact that the SARS-CoV-2 may be recorded in the saliva before lungs lesions appear. This explains the presence of asymptomatic infections. Therefore, it is possible to state that the salivary gland is not only the first access site for the SARS-CoV-2, but also one of the main reproduction sources, as it makes saliva highly infective and infecting [81,82,83]. Indeed, the high presence of corona virus SARS-CoV-2 in saliva of COVID19 patients reaches 91.7%, and from their saliva samples it is also possible to easily cultivate the virus in vivo [83,84,85,86,87,88,89]. 

There is a study analyzing the virus SARS-CoV-2 resistance to the internal surfaces and to the sun light. This study proved that the UV-C light (absent to the natural light) inactivates coronaviruses and that the UVB levels found in sun light may really inactivate the SARS-CoV-2 on surfaces, especially the dry virus on stainless steel specimens. This research provided the first evidence that sun light may quickly inactivate the SARS-CoV-2 on surfaces. Data suggest that the natural sun light may be also effective as a disinfectant for contaminated non-porous materials [90]. Researchers have also revealed that the simulated sun light is quickly able to inactivate the corona virus SARS-CoV-2 on specimens performed on stainless steel. The results of this study highlighted that 90% of the infecting virus was inactivated in a period of time consisting of 6.8 min in the saliva solution. The sun light necessary for those tests is similar to the summer solstice, in a not cloudy day. Researchers stated that the inactivation has been tested when the sun light levels were also lower [90,91].

### 1.3. Clinical Features

The article published by Doremalen has underlined chilblain, urticaria and tremor representing the new symptoms of the COVID-19 patient [92]. Moreover, some isolated cases were recorded, but only three cases in Madrid (two suspected and one confirmed) of herpetic-like vesicular lesions in the oral cavity with pain, desquamative gingivitis, and ulcers [93,94]. 

The involvement of the oral cavity in SARS-CoV-2 calls dentists to the front line once again in order to be able to perform an early clinical diagnosis.

The first SARS-CoV-2 found in the HU-1 patient of Wuhan, in the first days of December 2019, was perfectly adapted to the human being, as in the following months, despite the fact it reproduced itself trillions of times, the substantial and important mutation of genomic transcription did not occur, and the situation proceeded unchanged [19,20]. While for the SARS-CoVs there has been an increasing infectivity through meaningful mutations during the first months of the epidemic in the transmission from man to man, this is also the reason why scientists have had great difficulty in making a vaccine. Indeed, the SARS-CoV-2 has been a more stable virus than the SARS-CoVs since the beginning, which can also infect the first respiratory tracts, by encouraging the infection.

The affinity SARS-CoV-2 for its receptor in the host, ACE2, is 10–20 times higher than the SARS-CoV. Those two features make the SARS-CoV-2 much more infective than the SARS-CoVs. The SARS-CoV-2 virus has generally a reduced lethality but at the same time a higher risk of infection, it is more resistant to the survivors, while the SARS-CoVs virus is less contagious and very lethal, but it has been easily extinguished. Viruses with a lipid coating are generally more fragile. Thanks to its lipid coating, SARS-CoV-2 virus encourages the disactivating with the use of cleaning agents containing and all cleaning agents using hot water. However, in SARS-CoV-2, ORF8 and ORF3b proteins modulating antiviral and pro-inflammatory responses are very different from other similar SARS coronavirus, and this confers differences of pathogenicity and transmissibility. It has been shown that SARS-CoV-2 is extremely pathogenic, a powerful suppressor of the antiviral immunity, and it is also an activator of the pro-inflammatory response [95,96]. 

The cytokine storm syndrome leads to the interleukin release (IL) -6, IL-1, IL-12, and IL-18 together with the tumour necrosis factor alpha (TNF-α) and other inflammatory mediators. The increasing lung inflammatory response causes an increasing alveolar-capillary gas exchange, making hard the patients’ oxygenation in severe patients. The collapse of lung walls and severe bilateral respiratory insufficiency occur, as well as organs lesions with severe functional deficits. Severe lymphopenia and eosinopenia cause a decay in antiviral immunity. The recommendation is early screening for inflammatory markers, Ferritin, c-reactive protein (CRP) and D-dimer. Helper cells of type 1 mediate the delayed inflammatory response causing IL-6 activation and other pro-inflammatory cytokines. If it is not treated, the inflammatory reaction may lead to severe lung lesions [97,98,99,100,101,102,103,104,105,106,107,108].

The insignificant increase of serum markers before starting the treatment with hydroxychloroquine and azithromycin may lead to deleterious adverse effects. Moreover, it may be appropriate to make a differential diagnosis with active tuberculosis and active fungal infections [97,98,99,100,101,102,103,104,105,106,107,108].

The key role of IL-10 is to modulate the hyper-inflammatory response as well as the regeneration of damaged tissues in COVID-19 affected patients. The use of IL-10 tends to finalize the block process of IL-6 by the Tocilizumab and avoid the formation of damaged interstitial lung tissues in fibrotic tissue [99].

IL-10 is one of the last potential biologic therapeutic agents. It is able to regulate the functions of lymphoid and myeloid cells, IL-10 has a great inflammatory activity both in vitro and in vivo. 

IL-10 has been identified as a potential therapy for inflammatory diseases involving the helper 1 of type T (Th1) and the responses to macrophages. It is to be noted the occurrence of the secondary bacterial pneumonia during or immediately after the COVID-19 infection, determined by a complex interaction between the viruses, bacteria, and host [109].

The host remains more susceptible to bacterial infections even after several weeks from the elimination of the virus, this greater susceptibility is due to an increased viral virulence, indeed the infection increases the adherence and even for the following colonization with bacterial respiratory pathogenic agents. Bacteria adhere to the basal membrane after the interruption of the epithelial layer by air because of the cytopathic effect of the virus. This is caused by the greater adherence and it is due to the over-regulation of the receptors involved in the attack of bacteria. The SARS-CoV-2 alters the host’s immune response to the successive bacterial solicitations by getting more sensitive to the bacterial components, as the staphylococcal enterotoxin B and LPS. Cytokines, such as IFN-γ, TNF-α, and IL-6, are synergistically upregulated by the staphylococcal enterotoxin B or LPS during influenza infections. These data show that the virus SARS-CoV-2 significantly modifies the full immune response to the bacterial infections in a singular and atypical way. The process through the virus modulates the full immune response to the lung bacterial infections must necessarily be focused upon [110].

In vivo, most part of the tissue’s destruction is caused by an inflammatory response which is extreme and not-regulated, mainly neutrophil which, if not controlled, generates irreversible damage to tissues by decreasing the protective and regenerative dynamics [85,111,112]. 

The deficiency of IL-10 allows for the uncontrolled increase of IL-6, a phenomenon which mainly occurs in patients with comorbidity, immunosuppressed patients, diabetics, debilitated, elders, and patients deficient in vitamins, especially vitamin D [113,114,115,116,117].

Experiments on knockout deficient IL-10 mice spontaneously develop inflammatory syndromes as Irritable bowel syndrome (IBS). IL-10 in lungs was shown to be expressed by alveolar macrophages and stimulated by bacterial lipopolysaccharide (LPS), TNF-a. Regular bronchial epithelial cells produce anti-inflammatory cytokines interleukin-10, regulated by cystic fibrosis [97,118,119].

The average age of 2019-nCov infected patients is 55.5 years, while for mortality (case fatality rates- CFR), the age is 75 years, and it gets higher in the 80s age group. The number of deaths is higher present in the elder population with comorbidity and it enforces the key role that the Immune system played in the control of persistency of the SARS-CoV-2 virus. The decline of immunity occurs in the aging process, so the SARS-CoV-2 virus may access the respiratory tract in elder patients more easily [120,121].

Men are more affected than women (67%) as there are more smokers among men and because the woman immune system has a better antibody response linked to the X chromosome [122,123]. 

Diseases such as diabetes (7.3%), chronical respiratory infections (6.3%), cardiovascular problems (10.5%) hypertension (6%), and tumours (5.6%) are comorbidities constituting a high risk of infection [8,124].

Some studies on the ongoing of infections of babies, according to some recent epidemiological data coming from Norway, Iceland, South Korea, and China, even if analyzed on different sized samples, all confirm the same infection rate, namely that babies represent 1–5% of the infected population, and most of them are asymptomatic or show a slight or moderate symptomatology that is higher in the male population. A big percentage of babies (90%) with severe evolution of the disease interests the age group of 0–2 years. The presumed less occurrence of infection on babies is linked to the structural and functional immaturity of the cellular receptor ACE2 by offering a less affinity to the virus spike. In contrast on what happens to infected adults, most of infected babies seems to have slighter clinical course. Frequent asymptomatic infections classify the infected babies as asymptomatic infected patients [125,126,127].

The early diagnosis together with prevention methods (social distancing, use of personal protective equipment, such as face masks and wash handing with alcohol solutions) are important to contain and contrast the 2019-nCoV spreading. The 2019-nCoV contagion mainly occurs through saliva droplets and nasal secretions, and tears in less measure in faeces, urine, sperm, and blood [26,67,85,86,87,112].

## 2. Routes of Transmission for the SARS CoV 2

**Figure 4:**
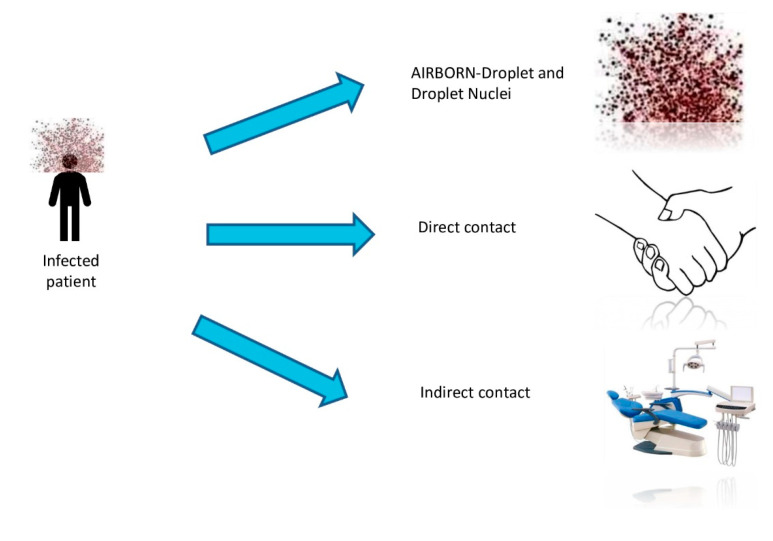
The transmission routes of COVID-19 (figure drawn by Giovanna Dipalma).

As dentists work in contact with patient, we have to know very well the possible paths of transmission of this virus. We already know that virus has several paths. First of all, there is the direct way from one person to another, by coughing, sneezing in the proximity of another subject, and by inhaling infected droplets (Flugge’s droplets). Another way of transmission is by contact with oral and nasal mucosa or conjunctival mucosa after touching an infected surface, as presented in Figure 4.

The way of contamination is by respiratory droplets that are considered to be particles that are made of water and have the ability to fall to the ground. They have a diameter bigger than 5µm. This type of droplets can be produced by breathing, talking, sneezing, coughing, or vomiting, or they can appear artificially being produced by aerosols generated during medical procedures, flushing toilets, touching of surfaces, or other domestic activities [128,129].

It is transmitted both by air, through the breath, and by direct contact with infected surfaces, including entrance via respiratory airways, mouth, nose, eyes. It has been shown that the virus enters through the eye conjunctiva, touching infected surfaces, and then bringing the virus to the face and mouth, as well as through the air conditioning that collects the virus from the air droplets produced by nebulization during dental operations). Hence, droplets containing the virus are collected by air conditioning and then are put back in the same environment, exiting the air conditioner at a minimum distance of eight meters, which is very dangerous compared to the two meters recommended for spacing [129,130,131].

Another route of transmission documented is through contact. All the personal items of the infected patient and the immediate environment can be considered as a potential medium for the transmission of the virus even by the indirect contact with those [9,132,133].

To check this hypothesis, a surgical mask was checked in order to determine the level of contamination and the results showed the presence of the virus on the outer layer even after a period of seven days [134].

### 2.1. Air Transmission

The virus does not travel in the outdoor air alone, but is conveyed through the droplets (Flügge’s droplets) issued by a COVID-19 patient who, through sneezing or coughing, reaches over two meters away. Flügge’s heavier droplets immediately fall to the ground due to the force of gravity and pollute the most contiguous surfaces to the infected patient, while the light ones, called aerosols, travel as in a gas cloud for which they are certainly transported further away (even several meters) and for more time [135,136].

### 2.2. SARS-CoV-2 Diffusion in Hospital

The risk of infection from SARS-CoV-2 can be found in any enclosed environment in which there are symptomatic patients, the hospital is a high-risk environment, as well as dental practices where water-cooled rotary and ultrasonic instruments are used. In the hospitals, a lot of manoeuvres and procedures that generate small droplets are performed: such as endotracheal intubation, open aspiration, administration of nebulized treatment, manual ventilation before intubation bronchoscopy, open aspiration, administration of nebulized treatment, disconnection of the patient from the ventilator, ventilation non-invasive positive pressure, tracheostomy and cardio-pulmonary resuscitation.

In environments where many patients are undergoing mechanical ventilation, constant air change becomes fundamental as the rooms become saturated with infected air. For this reason, patients hospitalized in intensive care or who are under ventilation must be kept in rooms with negative pressure. In a closed environment, there is certainly the air spread in the presence of the virus and the risk of contagion certainly enhances for people present in that closed environment. For this reason, constant air exchange is essential. An article published by Ong et al. concerns the search for sites of infection in closed environments [9,136,137].

As studies have shown, regarding the survival rate of SARS-CoV-2 on different surfaces, the management of medical waste can be considered as a crucial factor in order to control the spread of the infection. How we manage the medical waste should consider a manner that has to include various departments and individuals that have to collaborate in order to have specific protocols for every unit regarding SARS-CoV-2 [138].

## 3. Diagnostics

**Figure 5:**
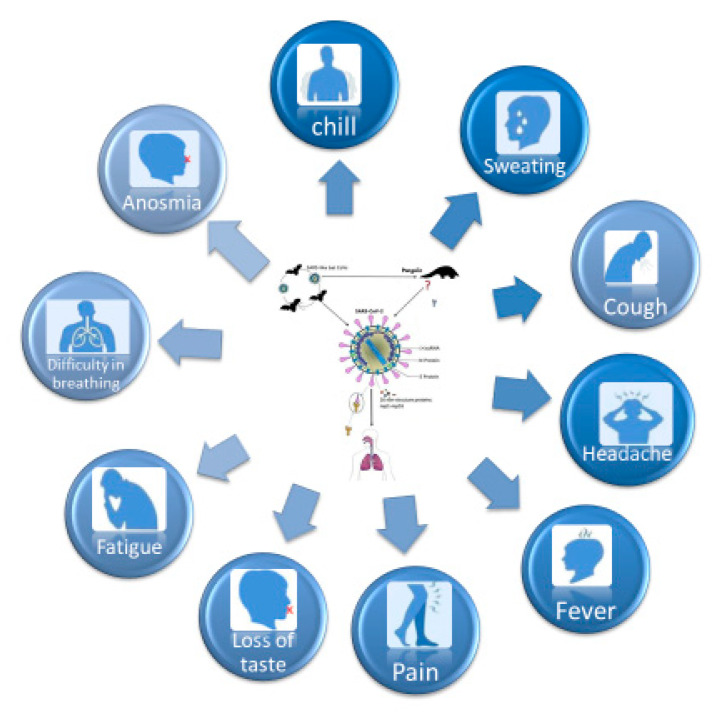
The specific Symptoms for COVID-19 (figure drawn by Giovanna Dipalma).

The specific symptoms identified are fever, dry cough and also other types of symptoms were identified like fatigue, anosmia, difficulty in breathing, loss of taste, muscle pain, confusion, headache, sore throat, diarrhoea, sweating, and chill, as shown in Figure 5.

The diagnosis of COVID-19 infection is based on methods of investigation such as the epidemiological data, clinical symptoms, computer tomography (CT) imaging, laboratory tests, and a history of travel to epidemic regions. The detection of the viral RNA can be confirmed via the re-verse transcriptase polymerase chain reaction (RT-PCR) test of upper or lower respiratory specimens or serum specimens. However, the false negative results of this test are not an indicator signaling the patient is virus free. Therefore, the most significant diagnosis tool is chest CT, as it shows a pure ground-glass opacity with consolidation in the posterior and peripheral lungs, which are signs of COVID-19 pneumonia. The chest CT is considered to be a more precise diagnosis tool that RT-PCR, regarding the sensitivity and the early detection of the presence of COVID-19 [139].

The aspects concerning the treatment are controversial and the randomized controlled trials did not validate any specific anti-SARS-CoV-2 treatment or vaccine. The management of the infected patients has been palliative until now, addressing the symptoms, and stabilizing patients ‘critical conditions towards full recovery. With the universal approaches toward COVID-19, including the identification of the source that determined the infection, measures taken in order to prevent the cross-infection, and the evolving diagnostic measures, more hospitals are receiving infected patients [140].

For dental professionals, the way they perform classic treatments has changed, and they have to adapt to new strategies in order to be able to perform dental treatments. For this reason, the dentist, before welcoming the patient into his office, must perform a telephone triage. During this triage, the patient is asked several questions as in this way the dentist is able to know if the patient has any specific COVID-19 symptoms, and then it is vital to know if the patient has travelled abroad, if he has been in contact with people that travelled abroad or if he has been submitted to a diagnostic buffer test for SARS-CoV-2. This telephone screening directs the practitioner for further, and if possible, greater precautions before the patient arrives at the dental office to perform the intended treatment [17].

In order to protect the patients needing dental care, we can use instruments that do not generate aerosols while performing treatments. For this reason, the use of lasers can be taken into consideration to replace the conventional use of hand pieces generating aerosols [141,142,143,144].

Until now the best technique for the clinical diagnosis of SARS-CoV-2 is based on the virus detection through a nasopharynx swab and sputum specimens and salivary tests, a technique established by the real time PCR and confirmed by the next-generation sequencing. This last method is useful to find future mutations of the SARS-CoV-2, but difficult to perform. The accurate diagnosis affected by SARS-CoV-2 is important to stop the global COVID-19 pandemic. However, the current diagnosis tests based on the RT-PCR are not so reliable (70%) as they are often not able to find several infected cases and must be performed in specialized laboratories. The delay in diagnosis has encouraged disease spreading. The diagnosis may be confirmed by a combination of clinical, epidemiological elements, as well as chest CAT, even if a negative RT-PCR may not exclude a SARS-CoV-2 infection [145,146,147].

The “Aldo MORO” University of Bari and Pham Chau Trinh University, Danang City (VIETNAM) of Vietnam have filled a license application for a diagnostic procedure for the multiple detection of pandemic Corona Virus. The test is based on the reverse transcriptase polymerase chain reaction RT-PCR, but for the RNA detachment of more viruses of the corona family, such as SARS-CoV-2, SARS-CoV, MERS-CoV, and HCoV by analysing respiratory tract samples (nasopharynx and oropharynx swabs) and lung sputum sampled by patients suspected of having COVID-19. As a result, there is a reliability of 99.9% in a very short time (less than 3 h) and it may be performed in any laboratory employing the RT- PCR technique. Usually, during the acute phase, the SARS-CoV-2 virus RNA is detectable in swabs. Positive or negative results constitute a response about the SARS-CoV-2 infection by referring to the clinical condition or asymptomatic patient. With a positive result, this technique does not exclude a combined bacterial infection or in association with other viruses belonging to the Coronavirus family. In this case, the disease cause is due to the result of the pathogen identified [148].

Mathur and Mathur suggest the use of antibodies as a screening test. Antibodies known as immunoglobins are searched for in blood as they mark the immune response to SARS-CoV-2 infection. In their opinion, these tests are indicated as screenings in areas with high prevalence, whereas in low prevalence areas, a high specificity test must be used [149].

Regarding the serological diagnostic performance, Zainol affirmed the evaluation of serology tests is not quite straightforward, because compared to PCR tests, these ones are capable of detecting the viral nucleic acid compared to the serological tests that are capable only to detect antibodies or host response to the infection [150].

The clinical assessment of the disease is classified in mild forms that present just mild symptoms, with no clinical signs of pneumonia, and the second stage is represented by the moderate form that presents respiratory symptoms accompanied by fever and with the presence of pneumonia, and the last type is represented by severe or very severe form [151].

## 4. Personal Protective Equipment

The dentists, but also the dental hygienists and dental assistants are potentially exposed to high degree of contamination with SARS-CoV-2 virus because they perform treatments that generate aerosols by using different devices such as turbines, micromotors and ultrasounds. It is very important to remember that the airways-oral cavity, nostrils, and eye conjunctiva are the access routes of SARS-CoV-2. 

Moreover, the salivary gland duct epithelium as well as the respiratory tract cells highly represent ACE2 receptor cells. Hence, while working in close proximity with the patient’s oral cavity, GDP is the most likely to contract the virus. In order to avoid this, extra caution and PPEs should be implemented [152,153].

**Figure 6:**
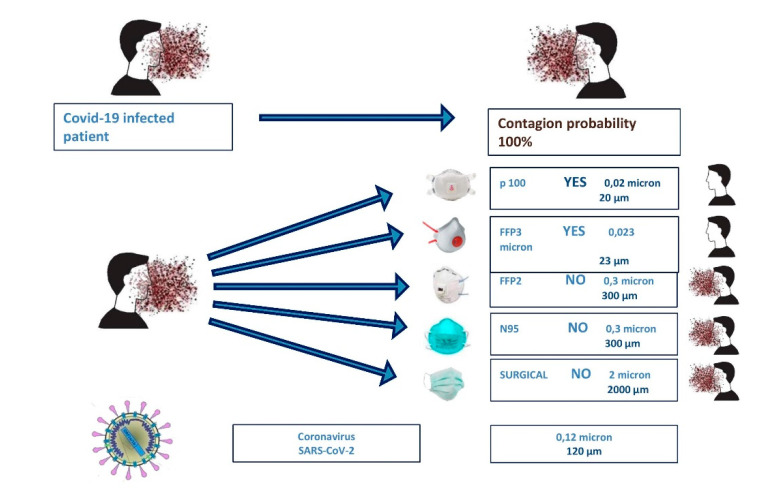
Different kind of masks and their effectiveness regarding the protection for infection with COVID-19 (figure drawn by Giovanna Dipalma).

All the professionals during this period have to take protective measures in order not to infect themselves and also their patients. For that the suggested equipment has to cover all the body and also the nose, eyes, and mouth. The protection for the mouth is considered to be the mask. In Figure 6, we present different types of masks in order to protect healthcare professionals [152,154,155,156,157].

**Figure 7:**
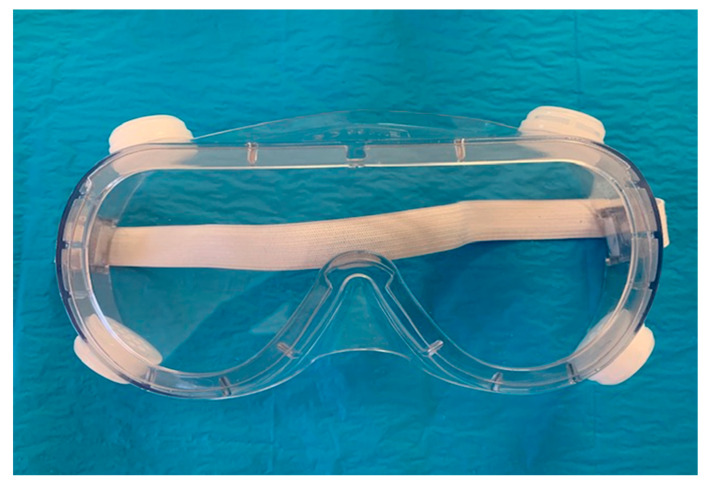
Eye protection glasses.

The protective equipment that is recommended by the World Health Organization (WHO) [158] includes the following:

1. Respiratory protection: N95 is highly efficient, while filtering masks with FFP2 (filtering face piece 2) or FFP3 (filtering face piece 3) are standard. Protective masks vary according to the production region; appropriate markings specification should be used for the assessment of the facemask. An orientation on the filtration potency of different masks is recommended by Domminiak et al. as shown in Figure 6:
  - FFP1 and P1: at least 80% filtration of all particles that are 0.3 μm in diameter or larger
  - FFP2 and P2: at least 94% filtration of all particles that are 0.3 μm in diameter or larger
  - N95: at least 95% filtration of all particles that are 0.3 μm in diameter or larger
  - N99 and FFP3: at least 99% filtration of all particles that are 0.023 μm in diameter or larger.
  - P3: at least 99.95% filtration of all particles that are 0.3 μm in diameter or larger
  - N100: at least 99.97% filtration of all particles that are 0.02 μm in diameter or larger
2. Eye protection, for example glasses and face shield as in Figure 7 [152,158,159].
3. Body protection such as waterproof, long-sleeved medical gowns and disposable head caps.
4. Hand protection such as sterile surgical gloves that should cover the cuffs of the gown [152,158,159,160,161,162].

**Figure 8:**
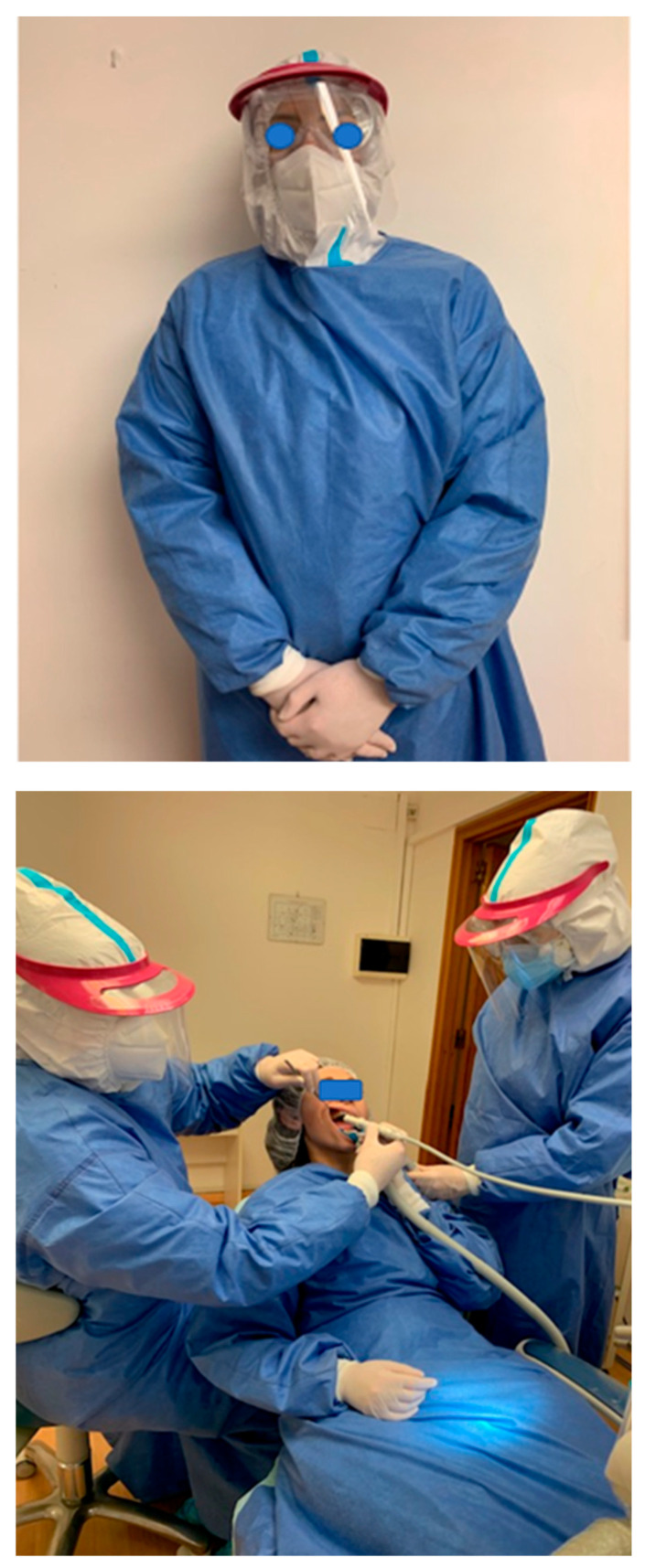
Personal protective equipment.

**Figure 9:**
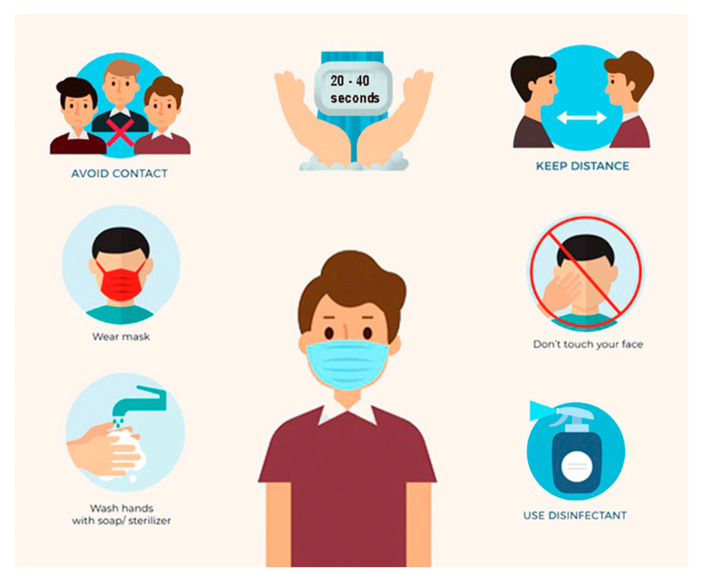
How to protect yourself from the COVID-19 (figure drawn by Giovanna Dipalma).

The FFP2 mask is one hundred times more effective, as a protective shield than surgical mask [153]. The protective equipment is represented in Figure 8 and the ways to protect ourselves from infection are presented in Figure 9.

A study performed by Scarano showed that the N95 masks produce a rise in the skin temperature and also discomfort while using them [163].

There are specific dental emergencies, according to the ADA, which “are potentially life threatening and require immediate treatment to stop ongoing tissue bleeding or to alleviate severe pain or infection.” Those conditions are uncontrolled bleeding, cellulitis, or a diffuse soft tissue infection with intraoral or extraoral swelling, which can potentially compromise the patient’s airway, as well as facial trauma [154].

The ADA [154] has added, as part of the emergency guidance, that urgent dental care should “focus on the management of conditions that requires immediate attention to relieve severe pain and/or risk of infection, in order to alleviate the burden on hospital emergency departments.” The conditions requiring dental treatments and should be managed as minimally invasive as possible, are as follows:

1. Severe dental pain due to pulpal inflammation
  1.1.2. Pericoronitis
  1.1.3. Post-operative osteitis or dry socket dressing changes.
  1.1.4. Localized bacterial infection resulting in localized pain and swelling.
  1.1.5. Tooth fracture resulted in pain or soft tissue trauma.
  1.1.6. Dental trauma with avulsion/luxation.
  1.1.7. Loss of temporary restoration due to tooth fracture, causing soft tissue irritation.

The non-emergency dental procedures, according to the ADA, include but are not limited to:

2. Initial or periodic oral examinations and recall visits, including routine radiographs.
  2.1.2. Routine dental cleaning and other preventive therapies.
  2.1.3. Orthodontic procedures other than those addressing acute issues (e.g., pain, infection, trauma).
  2.1.4. Elective extraction procedures.
  2.1.5. Restorative dentistry including, treatment of asymptomatic carious lesions.
  2.1.6. Aesthetic dental procedures [154].

**Figure 10:**
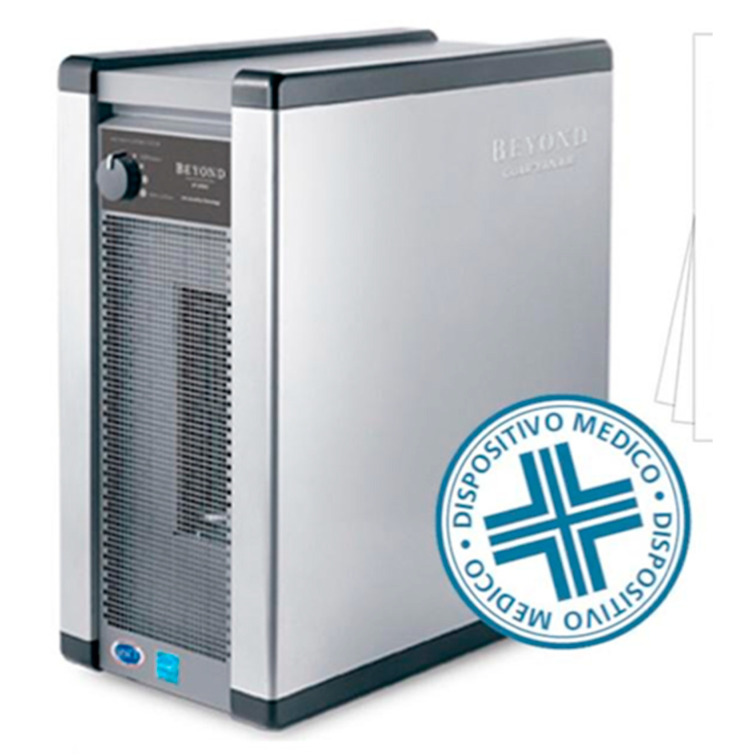
The sanitising system created by Nasa (https://www.activepure.com/italy-home/, https://beyond.vithagroup.eu/).

The air that is in the treatment room has a very high potential transmission pathway of the virus, if there is no adequate protocol for ventilation. The need to sanitize the environment between one patient and another one should be highlighted as ECDC recommends. For this reason, it is necessary to have rapid and effective sanitizing devices against bacteria and viruses. There are a great number of sanitizing devices, but many of them have limitations caused by the substances that are used for room sanitization. In fact, machines that use Ozone, chemicals, UV rays, or vaporized hydrogen peroxide involve some limitations, such as oxidation of the electronic boards and large sanitization times. These sanitizers described above necessarily involve a work break between one patient and another one of about an hour. For this reason, dental activity would have seriously slowed due to those long breaks, also causing economic damage. From a research of the devices, we discovered that only the machines that sanitize in the presence of operators and patients, during work with a higher standard HEPA filter inserted in sanitization devices, allow for operation in extreme safety without pauses between one patient and the next [152,154,155,156,157]. Such air sanitizing systems include those used with NASA space technology and certified with the ActivePure^®®^ name (registered trademark) Beyond (https://www.activepure.com/italy-home/, https://beyond.vithagroup.eu/). This device was created by NASA for spacecraft, transferred for terrestrial use by Aerus, tested in university laboratories, and is validated by all US certification bodies and by the Ministry of Health in Italy as a medical device. This device is shown in Figure 10.

**Figure 11:**
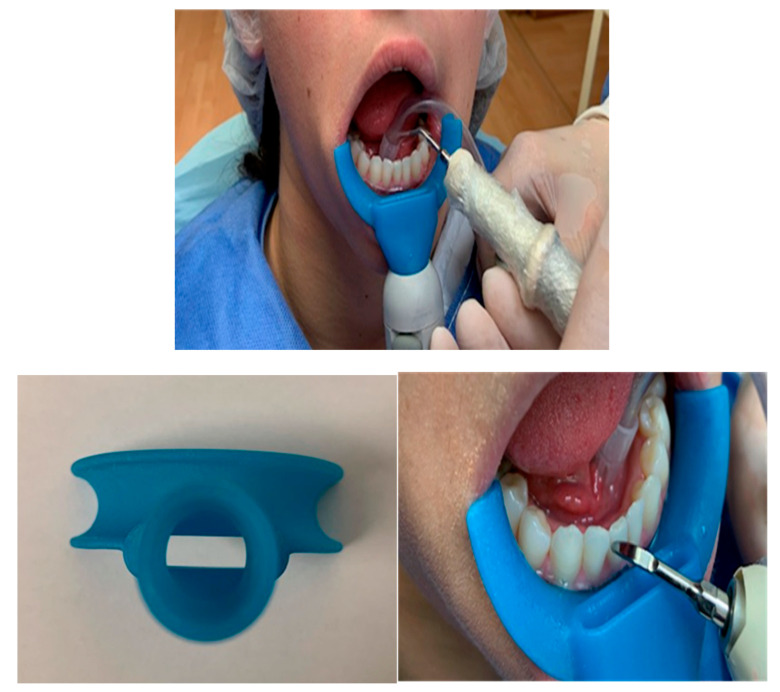
Power suction device.

In order to reduce the presence of aerosols, dental practitioners may use the double surgical suction in order to remove all the water from the oral cavity. On the market, there are also additional external suction devices to the dental chair, this is a third powerful suction transportable from one dental chair to another and additional to the two surgical aspirators already existent as represented in Figure 11.

It has been published that the use of a solution that contains oxidative agents, such as 1% hydrogen peroxide or 0.2% povidone-iodine, as an antiseptic mouth rinse that should be used at the beginning of every dental treatment, is considered to help decrease the bacterial loading and also the viral one (SARS-CoV-2) in the saliva [17,152,164].

## 5. The Resistance of the Virus on Surfaces

**Figure 12:**
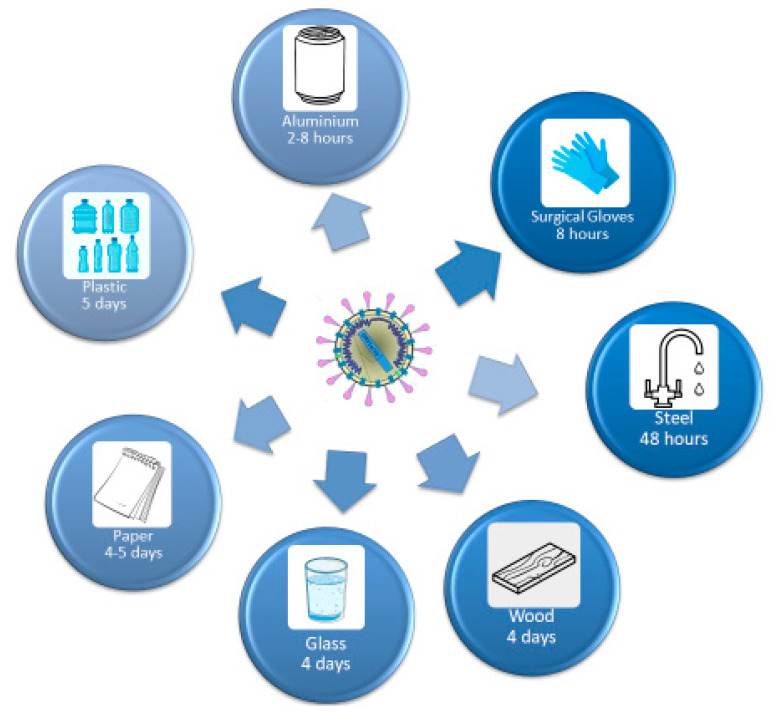
The persistence of SARS-CoV2 on different surfaces (figure drawn by Giovanna Dipalma).

The SARS-CoV-2 can survive up to three days on inanimate surfaces at room temperature, with a higher affinity for humid environments. In this context, all the staff at the dental practice need to ensure that all the inanimate surfaces should be disinfected with chemical detergents, which are recently approved for SARS-CoV-2, and a dry environment should be maintained to prevent the spread of SARS-CoV-2 as represented in Figure 12 [152,162,163,164,165,166,167]. 

There is preliminary evidence that environmentally mediated transmission may be possible, in particular, that COVID-19 patients could acquire the virus through contact with abiotic surfaces [92].

### 5.1. Disinfectants

The disinfection of the surfaces is very important and different chemicals can be used in order to perform the surface and equipment measures of asepsis. The chemicals that are recognized to perform this action are: Sodium hypochlorite, the phenolic compounds, disinfectants based on water or a combination of water and ortho-phenylphenol, tertiary amylphenol, or another type of chlorophenol, the O-benzyl, disinfectants at base of ethyl Alcohol, or isopropyl alcohol combined with ortho-phenylphenol or tertiary amylphenol, as well as disinfectants ethanol iodine complex based [69,168].

### 5.2. Sterilization Procedures and Methods of Medical Devices

For the sterilization of dental instruments, the known protocols have to be followed but with more caution and performed with autoclaves. Among the most used methods for the sterilization in health field, there are:

- With saturated steam.
- With ethylene oxide.
- With Hydrogen peroxide.
- Through peracetic acid solution [69,168,169].

#### 5.2.1. Sterilisation with Saturated Steam

This is a technique using the fluent steam or saturated (autoclave). It eliminates microorganisms through the denaturation of their proteins and other biomolecules. Sterilisation through autoclave is the most widespread being inexpensive and non-toxic, as well as thanks to its penetrating capacity. Sterilisation temperature (T) is of 1134 °C to the P of 2.1 bar. The time of minimal exposure of devices is from 5 to 7 min or 121 °C at P 1.1 bar. Time for this cycle (also defined autoclaving cycle) is from 15 to 20 min [69,168,169].

#### 5.2.2. Sterilisation with Chemical Means

The only chemical means still in use to sterilise is Ethylene oxide or ethoxide (EtO). It is used above all in hospitals because of its dangerousness. Indeed, it is an explosive gas and inflammable. The ethoxide has the feature of impregnate for long time the treated objects; in order to avoid body damage, before using these objects it is important to store them in ventilated areas or cabinets up to their complete sterilisation. Its action process is due to the alkylation, (that is to say the replacement of a hydrogen atom with an alkyl group) of sulfhydryl, amine, carboxylic, phenol, and hydroxyl groups and spore and vegetative cells. This process leads to the microorganism death. The contraindications of this method are:

- Cost.
- Toxicity.
- Long time for sterilisation and ventilation.
- It may be installed in an appropriate premise.
- Staff must have a license for toxic gas manipulation.
- It is reserved to sterilizable materials meeting the requirements in compatibility (modification of physical kind/levels of residual gas). It is not possible to perform the re-sterilisation of materials previously processed with gamma ray. Those limitations have led hospitals to an external running of the ETO sterilisation.

Another chemical mean used is the peracetic acid. The formaldehyde has been adopted in the past as chemical sterilising, but its use has been strongly limited by the law, having shown indications of carcinogenicity [69,168,169].

## 6. Treatment Ways Performed until Now for the SARS-CoV 2 Infection

The management of severe pneumonia determined by the SARS-CoV-2 is very challenging to all the medical teams and intensivists, due to lack of data, as it is a novel virus. Equally, it is very challenging for the infected patient, due to powerful medication protocol, which can cause burden on many organs, in terms of metabolism and excretion along with a series of side effects. At the present, until a vaccine can be procured, prevention always is the best approach for the COVID-19 pandemic [170,171].

Scientists, researchers, healthcare workers, and shareholders are investing all their efforts to produce a vaccine against SARS-CoV-2 to end this pandemic crisis. The current investigations are based on the immunological responses of T and B cells of infected subjects with SARS-COV, and compare the similarities between this virus and SARS-CoV-2. On this note, due to the genetic differences, only 23% and 16% of the known SARS-CoV T cell and B cell epitope of B cells mapping are identical to SARS-CoV-2 [172].

Several studies were conducted on different epitopes, in which major histocompatibility complex (MHC) class I and II epitopes are one of them. However, further experimental studies (T cell and B cell assays) are required to determine the potential of the identified epitopes to induce a positive immune response against SARS-CoV-2. This would help to further refine the reported epitope set, which is based on observed immunogenicity [173,174].

It is important to note that there is a lack of knowledge on the epidemiological and the biological properties, in view of vaccine production of the SARS-CoV-2 at the present time. There is also a lack of information on specific immune responses, against SARS-CoV-2 at this early stage, which represents a challenge towards vaccine development [172].

Searching for effective therapies for SARS-CoV-2 infection is a complex process. The disease can manifest, as upper respiratory tract disease and lower respiratory tract disease, which can be further subclassified as mild, severe, or critical, where patients should receive a support care to alleviate the symptoms and maintain the function of the vital organs in severe cases.

Great collaborative efforts are performed to discover and evaluate effectiveness of antivirals (remdesivir), immunotherapies (hydroxychloroquine, sarilumab), monoclonal antibodies and vaccines, which have rapidly emerged. However, these therapies have not been probably tested and the long-term effects and safety are still unknown [175]. Moreover, the demand for unproven therapies can cause shortages of medications that are approved and indicated for other diseases. Thereby, patients who depend on these medications for chronic conditions have been abandoned without effective therapies.

The association of virus with cytokine storm syndrome (CSS) is a fundamental challenge in its management, due to the severe consequences. The CSS is characterized by the release of interleukin (IL)-6, IL-1, IL-12 and IL-18, along with tumour necrosis factor alpha (TNF-α) and other inflammatory mediators. The increased pulmonary inflammatory response may result increasing in the alveolar-capillary gas exchange, resulted in difficulty in patients’ oxygenation with severe illness. Therefore, the recommendation is an early screening for inflammatory markers, ferritin, c-reactive protein (CRP), and D-dimer, and subsequently monitoring the trend of these markers. The goal is to initiate an early and aggressive immunosuppressive therapy. Initially, clinicians should rule out any other causes of an increased inflammation and deterioration of the clinical status such as: secondary infection, pulmonary embolism, or chronic obstructive pulmonary disorder (COPD) exacerbation. Patient with initial low ferritin level should not automatically be ruled out as a low risk for developing CSS, because low iron levels with or without anaemia can also cause reduced levels of ferritin. On the other hand, patient with high level of ferritin can be related to one of the following reasons: secondary infection, hepatitis B, hepatitis C, hemochromatosis, or other liver diseases [175].

This inflammatory response is a delayed response mediated by Type 1 T helper cells, causing an activation of IL-6 and other pro-inflammatory cytokines. On this note, the CSS progression can lead to acute lung injury if untreated.

Moreover, insignificant elevation of serum markers is not an indication for an immediate beginning of treatment with hydroxychloroquine and azithromycin, as this might lead to deleterious side effects. In this context, prior to any therapy, all patients should undergo laboratory evaluation for active tuberculosis disease or invasive fungal disease. Possible agents include IL-6 and IL-1 inhibitors or JAK inhibitors can be proposed, however, randomized clinical controlled trials to ensure the best treatment are not yet accessible. The treatment for severe pneumonia induced by SARS-CoV-2 is very challenging for both clinicians and patients, due to lack of data and the potent medications associated with the metabolism and excretion processes [175].

Some clinical trials have started in the UK, Germany, Switzerland, and USA, at the present time, toward vaccine development. There will not be such thing as a best treatment for COVID-19 until a vaccine is produced.

Currently, many drugs have been tried and used in the treatment of COVID-19 worldwide, but whether or not they are effective is subject to scientific evidence. However, these drugs are useful in a wide range of medical domains, especially in treating other viral infections effectively. These drugs are Chloroquine phosphate and Hydroxychloroquine sulphate, having a common mechanism of action, but caution is recommended. Although Hydroxychloroquine appears to have a higher efficiency. The viral entry can be blocked by inhibiting the glycosylation host receptors, proteolytic processing, and endosomal acidification, as well as by additional immunomodulatory effects inhibiting the cytokine production, autophagy, and lysosomal activity in host cells [176,177]. The association between hydroxychloroquine and azithromycin is showing tantalizing superior viral clearance infection, compared to the hydroxychloroquine monotherapy [108,178]. The general consensus is that an initial loading dose of 400 mg twice daily for one day, followed by 200 mg, twice daily for 5 days is an efficient dosage therapy. In terms of the adverse effects, both agents can cause prolongation of QT interval, which can lead to serious arrhythmia. Moreover, hypoglycaemia, neuropsychiatric effects and retinopathy are among the most frequent side effects. Notably, Azithromycin, as a monotherapy, can produce prolongation QT interval [171].

Hernandez et al. put in question the possibility of administrating hydroxychloroquine or chloroquine as a preventive treatment against COVID-19, but they concluded that the benefit harm ratio is too hard to asses [179].

Lopinavir/Ritonavir are drugs used for the treatment of HIV infection. They have shown to be a potential drug in treating novel coronavirus patients, via 3-chymotrypsin-like protease inhibition. After the endocytosis and uncoating of the virus, the single strand of RNA is translated into polypeptides, which undergo proteolysis to form non-structural proteins, and also these drugs act on the proteolysis link of this chain [180,181]. This medication has showed to have a significant efficiency, due to the multiple side effects (gastrointestinal intolerance, nausea, vomiting, diarrhoea, pancreatitis, hepatotoxicity, and cardiac conduction abnormalities). Nevertheless, for the other antiretrovirals, including protease inhibitors and integrase strand transfer inhibitors, further research consideration is required [171].

Ribavirin is a guanine analogue which inhibits the viral RNA-dependent RNA polymerase. This prevents the virus replication, which reduces viral load. Previous data regarding respiratory syncytial virus showed that inhaled administration of Ribavirin offers no benefit over enteral or intravenous administration. The adverse effects of this drug are hematologic (haemolytic anaemia) and liver toxicity. Ribavirin is also a known teratogen, and therefore it is contraindicated in pregnancy [182,183].

Remdesivir is a monophosphate prodrug, which undergoes metabolic process to an active C-adenosine nucleoside triphosphate analogue. At the present, this drug shows a promising potential for the treatment of COVID-19 due to its broad spectrum, potent in vitro activity against several CoVs [184].

Following its administration, reversible elevations of aspartate amino transferase/alanine amino transferase (ASAT/ ALAT) ratio were observed. The present posology is taken into consideration, as an initial loading dose of 200 mg and then 100 mg daily infusion. An initial dose is not indicated for patients with an estimated glomerular filtration rate less than 30 mL/min [171].

Favipiravir is a prodrug of a purine nucleotide, favipiravir ribofuranosyl-5′-triphosphate. The active form inhibits RNA polymerase; thus, it stops the virus replication. Evidence showed that an experience with influenza viruses and other corona viruses have for some time indicated different dosing variations. At this stage, the consensus is that doses at the higher end of the dosage range should be considered [185]. A loading dose is recommended (2400–3000 mg every 12 h × 2 doses) followed by a maintenance dose (1200 mg to 1800 mg every 12 h). The half-life of Favipiravir is approximately 5 h with mild adverse effects, however experience with higher doses is still limited [186].

As there is no proven specific therapy yet, an adjuvant therapy is still important and significant. In this category drugs to be considered are corticosteroids, anticytokine or immunomodulatory agents and immunoglobulin therapy [187].

Corticosteroids are used to reduce inflammation, especially in the lungs. However, it carries an increased risk of secondary infection and delayed healing from the virus infection. It is observed that corticosteroids do not necessarily improve the survival rate by extrapolating data from MERS, SARS as well as by other viral pneumonias, and also there is an increased risk to develop hyperglycaemia, psychosis and avascular necrosis, as well as, delayed viral clearance from the respiratory tract and blood. Interestingly, they are useful in bacterial infections and COPD exacerbation or refractory shock [171,188,189].

Anticytokine or immunomodulatory agents are based on the theory that they cause significant organ damage by an amplified immune response, therefore they are called “cytokine storm”. One of the key players seems to be IL-6. Therefore, treatment options have targeted this interleukin when Tocilizumab is used, which is a monoclonal antibody receptor antagonist. This drug seems to be highly effective, but the small sample size and the lack of a comparative group limits our interpretation. Moreover, Sarilumab is another IL-6 receptor antagonist, which is being studied [190].

Immunoglobulin therapy is based on the use of convalescent plasma or hyperimmune immunoglobulins. The concept of this therapy is to obtain antibodies from patient who completely recovered from the virus. Wang et al. study used the same concept in relation to other viral infections (H1N1) and showed to be successful. The maximum efficiency of this therapy occurs in the first 7–10 days of the infection when the viral load is at its peak and the innate immune response has not struck [191]. The use of extracorporeal membrane oxygenation (ECMO) for patients with respiratory failure, due to acute respiratory distress syndrome (ARDS) caused by SARS-COV-2 infection, is suggested. It is important to note that the use of this advanced therapy needs specific equipment, trained clinicians, along with a good protocol for maximum efficiency [192].

COVID-19 determines respiratory manifestations because of the production of pro-inflammatory cytokines (interleukin (IL) 1 beta and 6). IL-6 determines the interstitial pneumonia, and for its treatment during this period, Tocilizumab was used [99,193,194,195,196,197].

## 7. Conclusions

Our research has shown the high risk of infection which can occur in a dental practice, where there is vaporization and a great quantity of aerosol prompted through any dental operation, as well as the use of ultrasound and all rotating tools requiring water for cooling (turbines, micromotors, piezo-surgery). Our document provides some guidelines that doctors and dentists should scrupulously observe in order to prevent the spread of SARS-CoV-2.

Dental professionals should have knowledge regarding the safety procedures, the systems that can be used in order to purify the air, what measures they can take in order not to get infected, and how they have to perform dental treatments from now on. Because this information is changing, it will have to be updated with all the measures and protocols they have to follow.
